# Supplementary material for: Are carbon emissions trading and green financial instruments synergistic? -Comprehensive quantitative research based on content analysis
Source: PLoS One. 2024 Mar 7;19(3):e0298601. doi: 10.1371/journal.pone.0298601 (PMC10919601; doi:10.1371/journal.pone.0298601)
Supplement: S1 Data — (ZIP) [file pone.0298601.s001.zip › Data/地级市Z_F工作报告.zip!/║╙─╧/║╫▒┌╩╨/└┤╫╘╣1⁄2╓┌║┼í╛┬φ┐╦╩2╛▌═°í┐_2019.docx]

各位代表：

　　现在，我代表市人民政府，向大会报告工作，请予审议，并请各位政协委员和其他列席人士提出意见。

　　一、2018年工作回顾

　　过去的一年，面对复杂形势和繁重任务，在市委的正确领导下，全市人民坚持以习近平新时代中国特色社会主义思想为指导，深入贯彻党的十九大精神和习近平总书记视察指导河南时的重要讲话精神，全面落实省委、省政府各项决策部署，统筹推进稳增长、促改革、调结构、惠民生、防风险、保稳定工作，高质量发展实现良好开局，在中原更加出彩中走在前出重彩迈出了坚实步伐。

　　一是着力强化经济运行，主要指标平稳增长。坚持稳中求进工作总基调，深入贯彻新发展理念和高质量发展要求，创新出台系列政策措施，地区生产总值增长5.9%，规模以上工业增加值增长6.9%，固定资产投资增长7.7%，一般公共预算收入增长8.1%，社会消费品零售总额增长9.5%，居民消费价格上涨1.8%。新签约亿元以上项目169个，新落地141个，引进省外资金目标完成比例和增幅均居全省第三，货物贸易进出口目标完成比例和增幅均居全省第二。重点项目超额完成年度投资计划，第三产业投资增长21.8%、成为拉动投资的主要动力。被确定为全省普惠金融试点市，余额存贷比保持全省领先。为首批10家重点民营企业量身定制了“政策礼包”、配备了服务管家”，帮助企业解决了一批困难和问题，营商环境持续优化。经济技术开发区在国家考核中居全省第二，商务中心区晋位二星级，淇滨区创业创新园入选省级专业园区。

　　二是着力加快产业转型，经济结构更加优化。坚持以提高质量和效益为中心，深入推进供给侧结构性改革，百千亿级产业集群稳步壮大，因老工业基地调整改造力度较大等，被国务院表彰为落实有关重大政策措施真抓实干成效明显城市，产业转型升级示范区建设方案得到国家认可、获省政府批复。231个转型发展入库项目和144个绿色智能技术改造项目有序实施，新培育5个省级智能车间、智能工厂，全国唯一的镁交易中心获批运营，全省智能制造观摩排名豫北五市第一。签约落地京东（鹤壁）亚洲一号电商产业园、京东“互联网+”新经济以及机器人、无人机等一批新兴产业项目，互联网、大数据、云计算、人工智能呈现集聚发展良好态势。粮食总产保持稳定、责任制考核全省第二，全国首个粮油绿色高质高效示范基地建设通过验收，成为全省唯一达到国家农业现代化目标的城市。现代服务业呈现业态丰富、效益提升的态势，第三产业增加值增长6.9%。

　　三是着力深化改革创新，发展动能持续增强。坚持把改革创新作为推动发展的动力源泉。“放管服”改革取得新的进展，923项审批服务事项网上可办率和一网通办率100%，企业开办时间压缩到4.5个工作日，新登记注册市场主体增长18%以上。在全省第二个完成“僵尸企业”处置任务。煤炭行业去产能任务圆满完成。停滞半停滞项目和企业、闲置厂房楼宇盘活工作取得新的进展。“三块地”改革成效明显。农村土地承包经营权、宅基地使用权确权登记颁证基本完成，土地承包经营权抵押贷款试点成效显著，被确定为全国农村集体产权制度改革整体推进试点市。提前一年实现高新技术企业倍增计划，新创省级工程技术研究中心6家、为历年最多，省级科技成果转移转化基地实现零的突破，科技大市场建成投用，“豫创天下”、科技创新创业大赛取得优异成绩，被确定为省首批促进科技与金融结合试点市。高新技术产业增加值增速及占规模以上工业增加值比重、全社会研发投入强度增速均居全省前列，获国家科技进步二等奖1项、省科技进步奖4项和发明专利一等奖1项。

　　四是着力推进城乡建设，面貌功能逐步改善。坚持以人为核心，科学推进新型城镇化，有序推进乡村振兴。国土空间规划三项国家试点、城市双修、乡村振兴战略规划、主城区东部片区和浚县白寺片区规划等持续完善，老城区搬迁改造力度更大并获国家专项资金支持，城市可持续发展度排名全省第五。百城建设提质工程完成投资207亿元，其中海绵城市试点改造小区164个、道路130公里、管网120公里、水系34公里、公园绿地35处；清洁取暖试点年度考评全国第一，新增集中供热覆盖面积745.7万平方米，农户热源清洁化改造7.8万户，城镇和农村建设能效提升改造近80万平方米；拆除“两违”建筑48万平方米。节能减排示范连续3年考核优秀、累计获得上级资金15.6亿元，万元生产总值能耗下降5.1%，绿化造林12.3万亩，成功创建省级节水型城市。干线公路项目进展顺利，建设农村公路54.7公里，完成“四好农村路”年度任务，入选省“公交优先”示范城市创建名单。乡村振兴示范体系初步建立，新建改造城乡公厕147座，完成31个农村综合整治任务，农村垃圾治理全域达标，在《河南农业农村发展报告（2018）》中综合排名全省第二。预计常住人口城镇化率提高1.2个百分点以上。

　　五是着力打好三大攻坚，发展短板加快补齐。统筹做好政治安全、经济发展、社会稳定、自然灾害、公共事件、安全生产六大重点领域风险防控，非法集资、问题楼盘、危困企业、拖欠农民工工资、群体利益诉求等问题得到有效解决，在262个地级市综合信用排名中比2018年年初前移109个位次、升至第71位，政府债务规模可控，企业运行总体平稳，社会大局安全稳定。聚焦“2464”工作重点，大力开展季度专项活动，脱贫攻坚上半年重点工作核查被评为“好”的等次，全省产业扶贫现场观摩排名豫北五市第一，8573人实现脱贫，18个贫困村顺利摘帽。扎实开展蓝天碧水净土保卫战和环境整治风暴行动，细颗粒物（PM2.5）和可吸入性颗粒物（PM10）平均浓度分别下降6微克、4微克，优良天数增加8天，空气质量综合改善幅度全省第二，获“2+26”城市2017—2018年秋冬季大气污染综合治理考核优秀等次第七名、全省第一名，退出全国169个城市空气质量后20名，淇河水质保持全省首位，地表水责任目标断面平均浓度大幅下降、达到省定要求，饮用水水源地水质达标率100%。

　　六是着力保障改善民生，人民福祉不断增加。坚持以人民为中心的发展思想，持续加大民生支出力度，33项重点民生实事基本完成。居民人均可支配收入增长8.2%，就业再就业、转移就业等超额完成目标。社会保险实现扩面提标，新开工棚户区改造住房1784套、基本建成7351套、交付7996套。改扩建公办幼儿园6所，7个农村义务教育改薄项目全部竣工，完成23个扩充城镇义务教育资源项目，“大班额”问题得到缓解，校外培训机构治理基本完成。基本公共卫生服务综合评估考核全省第一，成为省“智慧卫监”试点市和妇幼健康服务信息系统试点市，城市公立医院综合改革获国家资金奖励，市人民医院成功创建“三甲”医院。民俗文化节、樱花文化节、淇河诗歌大赛、中原文博会、环淇河自行车赛等文体品牌更加响亮，基层综合性文化服务中心建成率88%，新增国家和省级传统村落各13个，辛村、鹿台考古发掘取得重大发现，豫剧牛派艺术和泥塑艺术荣登春晚舞台，在省文创之星、“群星奖”音乐舞蹈大赛中成绩突出。平安鹤壁建设扎实推进，扫黑除恶专项斗争、反暴恐斗争、反邪教斗争有序开展，社会治安、安全生产、信访稳定、食品药品安全等形势平稳，居民幸福感、公众安全感和法治环境满意度全省领先。

　　一年来，我们切实加强政府自身建设，坚决贯彻党要管党、全面从严治党方针和新时代党的建设总要求，坚定不移正风肃纪，中央巡视、中央环保督察“回头看”等反馈问题基本办结，市政府领导班子换届圆满完成，机构改革稳步推进；我们切实加强民主法治建设，自觉接受人大法律和工作监督、政协民主监督以及其他方面的监督，支持民主党派、工商联和无党派人士参政议政，办理人大代表建议和政协提案347件，是全省3个依法行政工作先进省辖市之一；我们切实加强精神文明建设，扎实推进社会主义核心价值体系学习教育，认真落实意识形态工作责任制，深化文明城市创建和农村精神文明建设，县级以上文明村镇超过50%，建成新时代文明实践中心（所、站）575个，“鹤壁好人”影响力持续提升；我们切实加强国防教育、国防后备力量及人民防空建设，支持军队改革发展，驻鹤人民解放军、武警部队和预备役人员、民兵为地方发展和安全稳定作出新的贡献，军民融合深度发展，连续五届被评为省双拥模范城。同时，民族宗教工作创新推进，妇女儿童、残疾人、红十字、慈善等事业健康发展，审计、外事、侨务、对台、史志、统计、档案、气象、地震、通信、援疆等工作得到新的提升。

　　以上成绩，是以习近平同志为核心的党中央坚强领导的结果，是省委、省政府和市委科学施策的结果，是市人大和市政协大力支持的结果，更是全市人民拼搏奉献的结果。在此，我代表市人民政府，向全市人民，向各民主党派、各人民团体和各界人士致以崇高的敬意，向关心支持鹤壁发展的各界朋友表示衷心的感谢！

　　在看到成绩的同时，我们也清醒认识到，经济社会发展还面临诸多困难和挑战。一是经济下行压力加大。受外部环境影响、自身问题叠加、结构矛盾凸显等制约，部分重点企业生产经营困难，平台和要素支撑能力不强，发展内生动力不足，主要经济指标增速放缓。二是转型任务依然艰巨。三次产业结构不优，城乡建设和民生领域欠账依然较多，科技创新和成果转化能力相对偏弱，财税和居民增收后劲不足，环境质量有待进一步改善。三是风险隐患不容忽视。不确定因素明显增多，市场风险、债务风险、信用风险、企业风险、金融风险、社会风险、安全风险等交织叠加，维护安全稳定的任务依然艰巨。四是自身建设需要加强。政府工作人员能力素质难以适应高质量发展要求，观念落后、作风浮漂、推拖庸懒、不作为慢作为等现象不同程度存在，一些领域不正之风和腐败问题时有发生。对此，我们一定高度重视、着力解决，努力在新的一年里把各项工作做得更好。

　　二、2019年工作安排

　　今年是新中国成立70周年，是全面建成小康社会的关键之年，也是创建党的建设高质量试点市和建设高质量发展城市的开局之年，做好各项工作意义十分重大。

　　政府工作的总体要求是：以习近平新时代中国特色社会主义思想为指导，全面贯彻党的十九大和十九届二中、三中全会精神及中央经济工作会议精神，持续落实习近平总书记视察指导河南时的重要讲话精神，深入学习落实省委十届六次、七次、八次全会精神，统筹推进“五位一体”总体布局，协调推进“四个全面”战略布局，坚持稳中求进工作总基调，坚持新发展理念，坚持推动高质量发展，坚持以供给侧结构性改革为主线，坚持深化市场化改革、扩大高水平开放，贯彻“巩固、增强、提升、畅通”的方针，深入开展三大攻坚战，持续推进“四个着力”、打好“四张牌”，统筹推进稳增长、促改革、调结构、惠民生、防风险、保稳定工作，进一步稳就业、稳金融、稳外贸、稳外资、稳投资、稳预期，提振市场信心，增强人民群众获得感、幸福感、安全感，着力以党的建设高质量推动高质量发展城市建设，保持经济持续健康发展和社会大局稳定，为率先实现全面小康打下决定性基础，谱写高质量富美鹤城建设新篇章，奋力在中原更加出彩的征程中走在前出重彩，以优异成绩庆祝中华人民共和国成立70周年。

　　经济社会发展的主要预期目标是：生产总值增长7.5%，第三产业增加值增长9%，规模以上工业增加值增长8%，全员劳动生产率增长7%，一般公共预算收入增长7.5%左右、税收占一般公共预算收入的比重达到72%，固定资产投资增长8.5%以上，社会消费品零售总额增长10%，居民消费价格涨幅3%左右。研究与试验发展经费投入强度增速高于全省平均水平，高技术产业、战略性新兴产业增加值分别占规模以上工业增加值比重与全省差距进一步缩小。常住人口城镇化率提高1个百分点以上，人均预期寿命持续提高，义务教育巩固率完成省定目标，医疗卫生、文化、体育等社会事业全面进步。单位生产总值能耗、主要污染物排放量完成省定目标。进出口保持平稳增长，实际利用外资完成省定目标，营商环境力争位居全省前列。居民人均可支配收入增长8%，城镇登记失业率控制在4%以内。

　　落实总体要求、实现预期目标，必须认真贯彻落实市委九届七次全会和市委经济工作会议精神，着力抓好八项重点工作：

　　（一）巩固良好发展态势，保持经济稳定增长。坚持精准出招应变，努力扩大市场需求，大力发展实体经济，确保经济行稳致远。一是深化开放招商。开展开放招商提升年活动，深度融入对接国家“一带一路”和省“五区一群”等战略，精心筹办参加第十四届豫商大会等招商活动，新签约、新落地亿元以上项目分别达到120个以上。进一步放宽外资准入限制，积极申建鹤壁综合保税区，加快跨境电子商务试验区建设，做大做强电子信息、镁精深加工2个省级出口基地。加快综合贸易服务平台建设，创建国家级电子信息外贸转型升级基地，服务贸易进出口、对外投资均增长10%以上。二是扩大有效投资。开展项目建设提升年活动，抓好102个市重点项目建设，完成投资375亿元以上，力争47个新开工重点项目10月底前全部开工，确保23个计划竣工重点项目11月底前全部竣工，新谋划重大项目300个以上。加快政府与社会资本合作项目、债券项目实施，带动扩大工业投资、民间投资，着力增加高技术、新兴产业等领域的投资。三是优化营商环境。开展营商环境提升年活动，组织实施营商环境核心指标对标优化行动和营商环境重点领域提升行动，积极创建全省营商环境示范区。加快清理违反市场原则、妨碍生产要素流动、影响企业活力的政策文件，实施统一的市场准入负面清单，消除民营企业在准入许可、工程投标、银行融资等方面的不公平待遇。构建亲清新型政商关系，完善党政领导与民营企业家恳谈机制、民营企业困难问题协调解决机制等，充分发挥企业“服务管家”作用、结对帮扶企业达到100家左右。依法打击侵害民营企业权益的各类违法行为，坚决制止对民营企业一切不必要的检查、评比、考核等活动。完善金融业发展规划和奖励优惠政策，加快普惠金融试点建设，实施“引金入鹤”工程，大力引进股份制银行，争取更多金融机构来鹤开展业务或设立分支机构。四是发展非公经济。引导企业聚焦主业、适度规模、规范治理、注重研发、提升品牌，加快建立现代企业制度，增强高质量发展的内生动力和适应新常态下市场经济的能力。加大民营企业家培养力度，遴选50名领军型企业家和50名新生代企业家开展高端培训。实施优质企业培育行动，打造一批“专精特新”“单项冠军”“小巨人”等企业，扩大“四上”企业数量。建立金融顾问服务团，深入企业开展巡回服务，帮助对接合适的金融产品，满足不同企业的融资需求。加强上市后备企业培育，力争主板、科创板上市实现突破。五是加强财源建设。加快财税大数据管理应用系统建设，实行税源清单管理，深入挖掘金融、物流、电商、建筑等领域的潜在税源，支持发展速度快、税收贡献多、综合效益好的企业做大做强。有序推进市以下财政事权和支出责任划分改革，全面实施预算绩效管理，提高收入质量和资源配置效率。

　　（二）深入开展三大攻坚，筑牢全面小康基础。把打好三大攻坚战作为破解瓶颈制约、补齐发展短板、建成小康社会的硬任务，深入谋划，全面发力。一是防范化解风险。加大互联网金融、小贷公司、融资租赁、保理公司等监管力度，扎实做好资金链出现问题企业的风险化解，深入开展加强诚信建设优化金融环境专项行动，继续推进不良贷款清收盘活、非法集资处置、恶意逃废银行债务行为打击等工作。加强政府债务管理和审计监督，规范政府举债融资行为，积极防范政府隐性债务风险。做好突发公共事件预防处置，防范社会风险。二是提升脱贫质量。把防止返贫摆上突出位置，围绕实现“两不愁三保障”，落实精准方略，强化产业支撑，建成240个以上脱贫攻坚项目，完成投资1.7亿元以上。加快改善贫困地区生产生活条件，实现所有贫困村通硬化路率、通邮率100%，完成所有贫困村饮水安全巩固提升工程建设任务，全面提升贫困村电网供电能力，所有贫困村生活垃圾治理达到省级验收标准。扩大电商扶贫站点覆盖率，争取70%以上的建档立卡贫困村设有扶贫站点。深入实施易地扶贫搬迁产业扶贫专项行动，力争所有搬迁户全面脱贫。继续做好残疾人等扶贫兜底保障工作。认真开展对口帮扶。三是抓好污染防治。严格落实党政领导责任、企业主体责任、部门监管责任，深入实施污染防治攻坚战三年行动计划和环境整治风暴行动，打好蓝天碧水净土保卫战，确保空气优良天数230天以上，可吸入性颗粒物（PM10）平均浓度每立方米103微克以下，细颗粒物（PM2.5）平均浓度每立方米55微克以下，完成京津冀及周边地区秋冬季大气污染综合治理任务；地表水达到或优于Ⅲ类水质断面比例、劣V类水质断面比例总体达到省定目标，淇河保持Ⅱ类水质，饮用水水源地水质达标率100%；农用地污染耕地和建设用地污染地块安全利用率保持100%。

　　（三）着力优化产业结构，促进经济提质增效。瞄准高质量发展方向，深化供给侧结构性改革，加快建设现代化经济体系，推动龙头骨干企业扩规模、提质量、增效益，积极创建全国产业转型升级示范区。一是深化“三去一降一补”。严格执行环保、能耗、质量、安全等标准，推动落后产能依法依规退出，严控过剩行业新上产能，推动更多产能过剩行业加快出清。完善住房市场体系和住房保障体系，努力扩大住房消费，保持房地产市场健康发展；继续推进停滞半停滞项目和企业、闲置厂房楼宇盘活工作；加快推进原址配建公租房工作，完善住房租赁补贴制度，完成棚改安置房逾期未交付使用项目整改任务。拓宽融资渠道，扩大融资规模，优化融资结构，有效发挥各类发展投资基金作用。落实减税降费政策，清理规范收费项目，降低融资、用电、物流成本，让企业轻装上阵。聚焦交通、能源、水利、脱贫攻坚、农业农村、生态环保、社会民生等领域，实施一批补短板项目。二是改造升级传统产业。持续开展转型发展攻坚行动，加快绿色、智能、技术改造，实施100个以上重点技术改造项目，支持企业增品种、提品质、创品牌，着力壮大优势产业集群。汽车电子电器与新能源汽车产业，积极发展高端汽车电子、智能网联电子、新能源汽车及配套零部件等，重点抓好新能源智控、车联网影音娱乐系统、新能源汽车产业园等项目；清洁能源与新材料产业，聚焦化工新材料、精细化学品、清洁能源和新型建材，重点抓好年综合利用150万吨煤焦油、生物化工产业园、年产3万吨精细化学品等项目，加快建设国内一流的现代精细煤化工基地；绿色食品产业，围绕肉制品、面制品、调理快餐、休闲饮品，重点抓好60万吨玉米深加工、年产11万吨乳饮料等项目，打造全产业链可追溯绿色食品生产基地；镁精深加工产业，加快与新能源汽车、智能制造、国防军工等融合发展，拓展镁合金在电子信息、交通等领域应用，重点抓好镁基轮毂、模板、新材料等项目建设，充分发挥镁交易中心作用，打造国家金属镁产品质量提升示范区和镁专利导航综合实验区。同时，提质发展现代服务业，以专业物流、电商物流、快递物流等为重点，规划建设白寺物流产业园，加快推进京东（鹤壁）亚洲一号电商产业园、豫北煤炭物流储配基地等项目，搭建智慧物流公共服务平台，争创省级城乡高效配送试点城市，努力建设豫北重要物流中心城市；深入挖掘特色文化内涵，传承发扬民俗文化、淇河文化、殷商文化、军事文化、古陶瓷文化、牛派艺术，大力发展智慧旅游、全域旅游，推进文化和旅游深度融合，加强景区基础和配套服务设施建设，积极创建云梦山—古灵山、大伾山—浚县古城5A级景区，统筹推进传统村落活态保护和乡村旅游发展，打造南太行重要旅游集散地，建设全国宜游城市；实施健康养老“十百千”示范工程，推进医养融合、健康养老等项目，完善“互联网+医疗健康”服务体系，建成10个社区日间照料中心，打造国内中高端养生养老基地。三是培育集聚新兴产业。抓住新一轮科技革命和产业变革的重大机遇，培育发展人工智能、大数据、新能源汽车、数字创意等产业，加快建设具有较强竞争力的战略性新兴产业基地。提前布局人工智能产业，加快光电子产业链式发展，扎实做好863科技创新园、中国（鹤壁）机器人硅谷、无人机等项目建设，培育发展智能产品，推进产业智能升级，打造人工智能发展高地。认真落实《河南省5G产业发展行动方案》和电信基础设施共建共享要求，统筹基础电信运营商5G设施建设，加快推进商用产品开发应用，打造在全省具有重要影响的5G设施建设先行区、创新应用示范区和集聚发展热点区。编制大数据产业发展规划，组建大数据产业发展有限公司，积极推动政府和社会数据资源整合，加快构建大数据基础服务平台和大数据产业发展公共服务平台，充分发挥京东云（鹤壁）数字经济产业园、农信通中国“三农”云计算大数据中心等带动作用，培育壮大大数据产业集群，打造区域性大数据中心。同时，紧盯国家和省重点培育发展的其他战略性新兴产业，找准契合点及时跟进，抢占产业发展先机；放宽服务消费领域市场准入，推进先进制造业与现代服务业深度融合。四是夯实发展载体平台。推进功能区体制机制创新，适时启动部分产业集聚区和服务业“两区”规划调整工作，形成定位准确、权责明晰、高效有序、分工合理的运行格局。实施产业集聚区“升星晋位”行动计划，全面提升产出效率、创新能力、产业能级和绿色发展水平；聘请一流专家做好宝山园区现代化工产业、基础设施建设、安全保障等规划。实施服务业“两区”高质量发展行动计划，加快服务业专业园区规划建设，积极申建省级示范物流园区，推动商务中心区加快发展企业总部、金融保险和法律、咨询等服务，推进特色商业区培育专业服务、文化体验等新业态模式。

　　（四）统筹推进城乡建设，促进融合协调发展。深入实施新型城镇化和乡村振兴战略，扎实做好百城建设提质和“六城联创”工作，加快创建全国城乡融合发展示范区。一是优化国土空间布局。充分发挥规划的战略引领和刚性控制作用，统筹推进市县两级2019—2035年国土空间规划编制、百城建设提质（城市双修）、重点区域等规划编制工作，进一步优化全市生产生活生态空间布局。加快推进国土空间规划信息平台建设，为推进国土空间规划实施管理、国土空间用途管制、资源利用、“三块地”改革构建更加科学的空间平台。二是打造贯彻新发展理念城市典范。高标准建设现代化主城区，在提升高速公路西侧精细化管理水平的基础上，加快建设高速公路东侧高质量发展城市创新引领区，作为建设高质量富美鹤城的破题之作，先期启动一批重大基础设施和公共服务项目，早日在大数据产业培育、科技创新策源功能集聚、现代文化教育提质、创新创业纵深发展、高品质公共设施支撑等领域取得突破。加快老城区转型振兴，深入实施老工业区搬迁改造、独立工矿区改造搬迁、采煤沉陷区综合治理等工程，建设全国老工业城市循环经济发展示范区。实施县域经济对标晋位行动计划，支持浚县发展现代农业、绿色食品、现代家居产业，打造国家级文化旅游名城；支持淇县发展多链条食品产业和综合性电商物流、冷链物流等现代服务业，加快特种尼龙产业园建设，打造国家级全域旅游示范县和全省纺织服装产业名城。规范引导特色小镇、特色小城镇建设。有序推进农业转移人口市民化。三是持续推进百城建设提质。把百城建设提质工程和文明城市创建有机结合起来，坚持宜居与宜业、地上与地下、硬件与软件、新区与老区“四个统一”，做好以绿荫城、以水润城、以文化城、以业兴城“四篇文章”，开展生态环境治污、交通秩序治堵、市容卫生治脏、公共服务治差“城市四治”，扎实推进城市规划、建设、管理、经营高质量，重点抓好生态水系、公园游园、公共停车场、背街小巷整治、老旧小区改造、城区亮化等432个项目，完成投资260亿元以上。加快推动日凤线、鹤辉高速、107国道东移等项目，完成乡道500公里和重要村道“四好农村路”建设任务。积极创建省“公交优先”示范城市。四是扎实开展“六城联创”。以创建全国文明城市、国家环境保护模范城市、国家生态文明建设示范市、国家生态园林城市、全国健康城市、全国双拥模范城为抓手，进一步优化文明环境、卫生环境、生态环境、健康环境、社会环境、人文环境，力争河南省文明城市、全国双拥模范城创建成功。持续提升城市精细化管理水平，逐步完善城市管理政策体系，加快构建权责明晰、服务为先、管理优化、执法规范、安全有序的城市管理体制，加强对环境卫生、交通秩序、集贸市场、街景市容、道路沿线、城中村、城乡接合部等重点难点问题集中整治，推动城市管理走向城市治理。五是深入实施乡村振兴战略。加快编制实施乡村振兴战略规划和专项行动计划，完善政策、扎实推进乡村振兴4个示范带、1个示范县、10个示范乡镇、83个示范村建设，积极推动重点镇争创全省百强乡镇，统筹推进城乡一体化示范体系、美丽乡村示范带、古村落古民居保护等工作，探索走出一条具有鹤壁特色的乡村振兴之路。深入实施乡村振兴“十大工程”，加快完善现代农业生产体系、产业体系、经营体系，做好整建制国家现代农业示范区、全国粮油绿色高质高效示范市、全国畜牧业绿色发展示范市、全国农业农村信息化示范基地、国家食品安全城市创建工作。坚持质量兴农、绿色兴农，推进种养业和绿色食品产业转型升级，新发展优质小麦20万亩以上、优质花生20万亩以上、优质林果2万亩，大力发展优质肉牛、优质奶牛、优质肉羊、优质家禽和特色养殖业。加大无公害农产品、绿色食品、有机农产品和农产品地理标志认证力度，新认证绿色食品10个以上、地理标志农产品2个以上。丰富创新农村一二三产业融合发展模式，着力壮大农业产业化集群、培育新型农业经营主体、强化农业科技支撑、发展新型农村集体经济，推动农业由总量扩张向质量提升转变。抓好污水垃圾处理、“厕所革命”、畜禽养殖等废弃物资源化利用、村容村貌提升等工作，打造一批“四美乡村”“美丽小镇”“五美庭院”，加快补齐农村人居环境和公共服务短板。塑造乡风文明，开展移风易俗。加强农村基层组织建设，推进乡村人才振兴。

　　（五）改善生态环境质量，共建共享美丽家园。深入贯彻习近平生态文明思想，加快构建现代化生态体系，积极创建全国全域生态环保示范区，叫响生态环保品牌。一是推动绿色城市建设树标杆。巩固提升国家森林城市、国家园林城市创建成果，编制《森林鹤壁生态建设规划》，持续推进国土绿化提速行动、山水林田湖草生态修复工程、国储林项目建设、非煤矿山整治等工作，新增造林绿化面积12.7万亩，建成5个森林特色小镇、50个森林乡村。严格落实河长制，大力开展水资源利用、水生态修复、水环境治理、水灾害防治“四水同治”，加快推进跨河流生态补水、南水北调调蓄水库等项目，提升淇河、卫河、汤河、共产主义渠等整治效果，逐步消除城区黑臭水体。二是推动低碳城市建设提层次。巩固提升国家节能减排财政政策综合示范市建设成果，严格控制煤炭消费总量、力争全年下降5%。加快北方地区冬季清洁取暖试点市建设，大力发展空气源热泵、生物质等清洁能源供热，力争实现农村“气做饭、电取暖、散煤清零”，确保清洁取暖建设保持全国领先。探索建设“步行城市”，持续推进产业低碳化、交通清洁化、建筑绿色化、服务集约化、主要污染物减量化、可再生能源利用规模化。三是推动循环城市建设上水平。以产业集聚区为载体实施产业园区循环化改造，加快静脉产业园建设，打造资源循环利用基地。做好总投资10亿元的垃圾焚烧发电、蔡庄垃圾处理厂垃圾坝加固增高、建筑垃圾处置与资源化利用等13个项目建设，不断提高垃圾分类处置能力和再生资源回收利用水平。四是推动海绵城市建设成品牌。把海绵城市建设理念贯穿城市发展各领域和全过程，新建区域全部按海绵城市要求设计施工，因地制宜搞好建成区海绵城市改造。扎实做好海绵城市终期考核验收工作，及时总结推广试点经验，打造在全国叫得响、能示范、可借鉴的“鹤壁模式”。五是推动节约城市建设增效益。持续提升节能、节水、节地、节材、节矿水平，积极创建国家节水型城市，抓好河南省装配式建筑示范产业园等项目建设。广泛开展绿色机关、绿色家庭、绿色学校、绿色社区等创建行动，建设资源节约型和环境友好型城市。

　　（六）持续深化改革创新，加快转换发展动能。坚持用改革创新的办法破解发展中的突出问题，不断提升改革成效和创新质量。一是加大力度深化改革。全面跟进落实中央、省改革部署，谋划推进好自主改革事项，着力破除体制机制障碍。深化“放管服”改革。深入推进“一网通办”前提下的“最多跑一次”改革，做好信息系统整合和数据共享，完善“互联网+政务服务”平台，推动企业和群众办事线上“一网通办”、线下“只进一扇门”、现场办理“最多跑一次”。深化商事制度改革，继续推进“多证合一”、企业登记全程电子化、外资企业“一口办理”等改革，扎实做好“证照分离”、扩大电子营业执照使用领域、提高企业名称登记效率等工作，进一步压缩企业开办时间，整合商事登记模块，完善市场主体简易注销登记制度。

　　深入推进“双随机、一公开”监管，加强涉企信用信息归集共享和失信联合惩戒。围绕实现“拿地即可开工”，大力推行投资项目审批“容缺办理”“多评合一”等模式，力争项目从立项到施工许可全流程审批时限压缩至60个工作日以内。加快公共资源电子交易平台建设，推进远程异地评标和公共资源交易全流程网上运行。深化要素市场化配置改革。落实国家完善要素市场化配置的意见，建立健全常态化要素配置调整机制、合理化要素流动机制和市场化要素价格机制。健全生态补偿机制，深入推进用能权有偿使用和交易试点建设，加快碳市场建设。实施能效水效领跑者制度。以直接交易和增量配电市场化为重点深化电力体制改革，扎实推进天然气、城镇供水、农业水价、交通运输等改革。深化三块地”改革。以城镇规划建设用地、农村耕地和乡村建设用地三块地”改革为突破口，强化对市本级土地的统一储备、统一“熟地”供应，加快耕地后备资源开发和农村土地信托平台建设，加大闲置低效用地处置力度，做好乡村建设用地、工矿废弃地复垦、“大棚房”清理整治等工作，充分释放土地资源开发利用潜力，推动资源向资产资本转化。深化农村综合改革。继续推进农村承包地“三权分置”改革，探索农村宅基地改革。加快农村集体产权制度改革，在全面完成清产核资任务的基础上，有序推进经营性资产股份合作制改革。加快建设县乡农村产权交易市场，引导农村产权规范流转交易。持续深化农村土地征收制度改革和农村集体经营性建设用地入市改革，加快建立城乡统一的建设用地市场。深化农村金融改革，强化农业保险保障作用。持续推进水利、供销社、农场、林权等改革。同时，抓好政府机构、教育医疗、警务管理、城市综合执法等改革。二是全面提升创新能力。以创建国家创新型城市为目标，积极争创国家可持续发展议程创新示范区、国家知识产权质押融资试点城市，争取列入郑洛新国家自主创新示范区辐射区、辐射点。大力实施重点企业培育工程，力争培育创新龙头企业2家，新增高新技术企业和科技小巨人（培育）企业10家；加快推进大中型工业企业研发平台全覆盖，新建国家、省级创新平台10家以上；申报实施“兴鹤聚才千人计划”，培养一批创新引领型人才；通过引进、联合共建等多种形式培育一批新型研发机构；强化企业孵化平台支撑，培育一批管理和服务水平高、运行效果好的科技企业孵化器、众创空间和星创天地。做好省科技与金融结合试点市建设，谋划推进一批重大创新专项，突破制约产业发展的核心关键技术。完善财政科技投入政策体系，引导企业加大研发投入，着力增加全社会研发投入。落实“三评”改革、科技成果评价改革和科技成果所有权改革等支持创新政策，发挥科技大市场作用，积极发展科技服务业，建立促进科技成果转化的资金支持机制，推动人才、成果、金融等创新要素和产业加速融合。建立试错、容错、纠错机制，让创新者敢于创新、安于创新、乐于创新。三是加快完善制度体系。扎实推进全国质量强市示范城市建设，加快构建高质量发展指标体系、政策体系、标准体系、统计体系和绩效评价、政绩考核办法，创建全省高质量发展体制机制示范区。在创新、协调、绿色、开放、共享发展和群众主观感受等方面，设置联系紧密、可操作性强的分领域指标和差异化指标，科学设置考核评价体系，创新考核评价方式。

　　（七）加快发展社会事业，优化公共服务供给。坚持抓重点、强弱项、提质量，持续提高公共服务水平。一是坚持教育优先发展。继续实施学前教育三年行动计划，促进学前教育普及普惠发展。推进城乡义务教育一体化发展，加快农村寄宿制和小规模学校建设，加大集团化办学等教育教学改革力度。有效提高全市教师编制使用效益，盘活优化各类教育资源，规范校外培训机构，持续解决中小学课外负担重、“择校热”、“大班额”等突出问题。改善普通高中办学条件。加快职业教育发展，持续推进产教融合、校企合作，推进鹤壁能源化工学院转隶、鹤壁汽车工程学院升本等工作。办好河南理工大学鹤壁工程技术学院。加强教师队伍建设，改善教师工作生活条件，提高山区农村教师生活补助标准。加强综合治理，维护校园安全。健全学生资助制度，使更多孩子享受到公平而有质量的教育。二是提高人民健康水平。巩固提升国家卫生城市建设成果，统筹抓好公立医院综合改革、分级诊疗、家庭医生签约服务、医联体建设、重点专科、全民健康信息平台建设等工作，支持社会办医，着力提升健康服务质量和水平。全面推进县域综合医改，加快推进县域医疗中心建设。加强生育全过程健康服务，提高妇幼保健水平。加快体育场、室外体育活动广场和体育馆、游泳馆（池）、全民健身综合馆“两场三馆”、城市社区“15分钟健身圈”等体育设施建设，开展全民健身活动，提高全民健康水平。加快创建国家食品安全城市，加强疫苗等药品安全监管，让群众吃得更放心、用药更安全。三是促进文化发展繁荣。践行社会主义核心价值观，大力弘扬焦裕禄精神、红旗渠精神、愚公移山精神。坚持文化强市，扎实推进淇河文化经济带、大运河文化带建设，深入实施文化惠民和公民道德建设工程，继续办好民俗文化节、樱花文化节、淇河诗歌大赛、中原文博会、环淇河自行车赛等系列节会赛事，提升博物馆、图书馆、文化馆等场馆开放服务水平，完成973个村（社区）综合性文化服务中心建设，抓好新时代文明实践中心建设，深化群众性精神文明创建。加强文物保护利用和文化遗产保护传承，谋划推进大赉店遗址公园和辛村国家遗址考古公园等项目。深入开展“扫黄打非”。着力打造新型主流媒体。提高网络综合治理能力。

　　（八）切实保障改善民生，不断增进人民福祉。坚持以人民为中心的发展思想，尽力而为、量力而行，精心做好各项民生工作。一是办好民生实事。牢固树立过紧日子思想，继续压减政府一般性支出，腾出更多财力物力，全力推进十件重点民生实事。（1）城镇新增就业2.97万人，其中失业人员再就业8600人、困难人员实现就业3300人。（2）实现7300名贫困人口稳定脱贫，12个贫困村脱贫摘帽。（3）基本建成棚户区改造安置住房3295套，基本完成农村低保户、分散供养特困人员、贫困残疾人和建档立卡贫困户4类重点对象农村危房改造清零目标。（4）改扩建公办幼儿园2所，新建1所、改扩建2所中小学校，建成农民体育健身工程50处、多功能运动场2个，举办城乡公益演出各100场，改造提升市文化中心，开工建设市老干部活动中心、老年干部大学。（5）新老城区快速通道改建工程建成通车、两侧高标准生态带建设任务完成60%，新增投放新能源公交车258辆，增设公交线路10条，建成富春江路公交停车场、鹤壁集公交停车场，新建电动汽车充电站5个、充电桩100个，新增公共停车泊位数3000个，打通东海路、珠江路、新闻巷、丁香巷、柳江路下穿高速铁路及护城河桥、花溪路下穿高速高铁等“断头路”。（6）新增集中供热覆盖面积300万平方米，新增农村清洁取暖2万户。（7）改造提升15所敬老院。（8）继续推进“厕所革命”，新建改造市区公厕48座、县域公厕34座，完成农村户用厕所改造6万户。（9）完成26个行政村电网改造升级。（10）完成盖族沟综合治理，建设城区北部生态廊道。二是稳定社会就业。把稳就业放在更加突出位置，实施就业优先战略，统筹推进高校毕业生、农村转移劳动力、就业困难人员、国企改革和去产能分流职工、退役军人、受中美经贸摩擦影响下岗返乡人员等重点群体就业，动态消除零就业家庭。积极推进公共就业服务专项活动和全民技能振兴工程，加大就业指导培训力度，深入开展失业保险援企稳岗“护航行动”和失业保险技能提升“展翅行动”，完成各类职业培训2.44万人次，新增农村劳动力转移就业0.9万人。三是健全保障体系。强化兜底保障功能，深入实施全民参保计划，以中小微企业、农民工、灵活就业人员等为重点对象，持续扩大社会保险覆盖范围。深化医疗保障制度改革，加强基金预算管理，完善总额预付制度，全面推行以按病种付费为主的多元复合式医保支付方式；稳妥推进委托商业保险经办改革，提高基本医保、大病保险、困难群众大病补充医保保障水平。加大社会保险风险防控力度，配合做好城乡居民基本养老保险基金省级管理、企业养老保险基金省级统收统支等工作，积极开展社会保险经办风险管理专项行动。继续调增企业和机关事业单位退休人员基本养老金，城乡居民基础养老金最低标准由每人每月98元增至103元，提高城乡低保、特困供养、孤儿养育等保障标准，严格落实特困供养、医疗救助、临时救助、残疾人补贴、高龄津贴、惠民殡葬等政策。真情关爱妇女儿童、残疾人，扎实做好福利慈善工作。做好盘石头水库第三期移民搬迁安置收尾工作。四是创新社会治理。学习推广新时代“枫桥经验”，全面加强基层社会治理。以群众满意为出发点和落脚点，深化信访案件集中研判会商机制，发挥信访工作联席会议作用，促进人民群众合理合法利益诉求及时、就地解决，实现信访总量明显下降、信访秩序明显好转、群众满意度提高。加强基层综治中心建设，创新网格化服务管理，完善基层治安防控网，夯实社会治理基础。加快立体化社会治安防控体系建设，推进“三级平台、四级联网”深度应用。完善“一评四会”基层矛盾纠纷预防化解等机制，高质量推进“一村（格）一警”工作，依法打击各种违法犯罪活动，推动扫黑除恶专项斗争向纵深发展，严密防范和严厉打击敌对势力、邪教组织、非法宗教活动向农村地区的渗透。健全公共安全体系，完善安全生产责任制，提升应急管理能力，坚决遏制重特大事故发生。扎实做好庆祝新中国成立70周年安保等工作，维护社会大局和谐稳定。

　　加强国防教育、国防动员和国防后备力量建设，支持国防和军队改革，支持驻鹤解放军、武警部队、民兵预备役部队和人民防空建设，进一步完善军民融合机制，深入开展双拥共建，加强退役军人服务保障体系建设，巩固军政军民团结。全面贯彻党的民族政策和宗教工作基本方针，加强民族宗教工作基层基础建设，促进民族团结、宗教和睦。支持工会、共青团、妇联等人民团体更好发挥桥梁纽带作用。做好审计、外事、侨务、对台工作。加强统计工作，搞好第四次经济普查。抓好档案、史志、气象、地震、邮政、援疆等工作。

　　三、加强政府自身建设

　　进入高质量发展新阶段，必须全面提高政府履职能力和服务水平，加快推进政府治理体系和治理能力现代化，努力建设人民满意的政府。

　　一是提高政治站位，抓好党的建设。增强“四个意识”，坚定“四个自信”，做到“两个维护”，在政治立场、政治方向、政治原则、政治道路上同以习近平同志为核心的党中央保持高度一致。深入学习贯彻党的十九大和习近平总书记视察指导河南时的重要讲话精神，坚持用习近平新时代中国特色社会主义思想武装头脑、指导实践、推动工作。落实市委“1567”党的建设高质量总体思路，为推动高质量发展提供坚强的政治保证。

　　二是弘扬法治精神，严格依法行政。加快法治政府建设，扎实做好立法、普法、政务公开等工作，在全社会营造尊法学法守法用法的良好氛围。坚持依法施政、依法行政、依法办事，规范行政执法行为，提高运用法治思维和法治方式推动工作的能力，努力做到严格规范公正文明执法。广泛听取民主党派、工商联、无党派人士和各人民团体的意见，提高决策科学化、民主化、法治化水平。自觉接受人大法律和工作监督、政协民主监督，主动接受社会和舆论监督，认真办理人大代表建议和政协提案。

　　三是践行为民宗旨，保持清正廉洁。把人民对美好生活的向往作为奋斗目标，政府要过“紧日子”、让群众过“好日子”，政府要习惯“快节奏”、让群众享受“慢生活”，政府的工作标准要越来越高、让老百姓的生活越来越好。坚决贯彻中央八项规定及实施细则精神，时刻防范、及时纠正“四风”隐形变异新动向新表现，推动政府系统党风廉政建设向纵深发展。严格遵守廉洁自律准则，筑牢思想防线，常态化制度化推进以案促改工作，自觉划清边线、不碰红线、守住底线，做到忠诚、干净、担当。

　　四是增强能力素质，锤炼过硬本领。提高敢于斗争的本领，加强斗争历练，永葆斗争精神，以“踏平坎坷成大道，斗罢艰险又出发”的顽强意志，应对好每一场重大风险挑战，做实做好改革发展稳定各项工作。提高驾驭经济的本领，自觉加强现代金融、数字经济、资本运作、项目策划等方面的学习，认真研究专业知识和政策走向，培养造就一支学习型、研究型、专家型的干部队伍。提高解决难题的本领，多到基层一线、项目现场，善于抓住问题本质、找准矛盾焦点，增强化解问题、解决难题的能力。

　　五是始终恪尽职守，激励担当作为。牢固树立正确的政绩观、事业观，深入整治不作为、乱作为、不想为、不能为现象，坚决反对空泛表态、应景造势、敷衍塞责、沽名钓誉等行为。认真做好思想解放大讨论活动，促使各级领导干部转观念、强思想、勇担当，以新思想统领大发展，以新理念引领大发展。坚持严管与厚爱结合、激励与约束并重，营造人人尽责、全民实干、奋发向上、担当作为的良好局面。

　　各位代表！实干书写精彩答卷，奋斗开创美好未来。让我们紧密团结在以习近平同志为核心的党中央周围，高举习近平新时代中国特色社会主义思想伟大旗帜，在省委、省政府和市委的坚强领导下，携手并肩，砥砺奋进，共同为率先实现全面小康、建设高质量富美鹤城而努力奋斗！
